# Diagnostic Value of Autoantibodies against Ezrin in Esophageal Squamous Cell Carcinoma

**DOI:** 10.1155/2017/2534648

**Published:** 2017-02-16

**Authors:** Lan Li, Ming Liu, Jian-Bang Lin, Xin-Bin Hong, Wen-Xia Chen, Hong Guo, Li-Yan Xu, Yi-Wei Xu, En-Min Li, Yu-Hui Peng

**Affiliations:** ^1^Shantou University Medical College, Shantou 515041, China; ^2^Department of Radiology, Cancer Hospital, Shantou University Medical College, Shantou 515041, China; ^3^Department of Radiation Oncology, Cancer Hospital, Shantou University Medical College, Shantou 515041, China; ^4^Institute of Oncologic Pathology, Shantou University Medical College, Shantou 515041, China; ^5^Department of Clinical Laboratory Medicine, Cancer Hospital, Shantou University Medical College, Shantou 515041, China; ^6^The Key Laboratory of Molecular Biology for High Cancer Incidence Coastal Chaoshan Area, Guangdong Higher Education Institutes, Shantou University Medical College, Shantou 515041, China; ^7^Department of Biochemistry and Molecular Biology, Shantou University Medical College, Shantou 515041, China

## Abstract

Esophageal squamous cell carcinoma (ESCC), one of the most common malignancies worldwide, is a highly aggressive and homogeneous entity occurring in esophageal squamous epithelium, and a reliable noninvasive test for early detection is needed. A recent study showed that serum autoantibodies against Ezrin could be detected in patients with pancreatic cancer. Here, we assessed whether autoantibodies against Ezrin could have diagnostic relevance for early ESCC. We analyzed autoantibodies against Ezrin in sera of 98 normal controls and 149 patients with ESCC. Ezrin autoantibodies levels were evaluated by enzyme-linked immunosorbent assay (ELISA). Results showed that higher levels of autoantibodies against Ezrin were observed in serum samples from patients with ESCC than in serum from normal controls (*P* < 0.0001). Based on a cutoff value of 0.319, the sensitivity and specificity of autoantibodies against Ezrin for diagnosis of ESCC were 27.5% and 95.9%, respectively. Compared with normal controls, the positive rate of autoantibodies against Ezrin was significantly elevated in patients with early-stage ESCC (*P* < 0.0001). Moreover, there was no significant difference of positivity of autoantibodies against Ezrin in ESCC patients categorized according to age, gender, tumor size, tumor invasion depth, tumor site, histological grade, lymph node status, or tumor stage. Our study indicates that the presence of autoantibodies against Ezrin is significantly associated with ESCC.

## 1. Introduction

Esophageal cancer is the 8th most common malignancy and the 6th leading cause of cancer-related mortality in the world [1]. Esophageal squamous cell carcinoma (ESCC) is the major subtype of esophageal cancer in China, which is one of the areas with the highest morbidity of esophageal cancer [2]. Incidences of esophageal squamous cell carcinoma (ESCC) have been reported to reach up to 100 cases per 100,000 annually in an area referred to as the “Asian esophageal cancer belt” (from northeast China to the Middle East) [2]. In spite of many advances in treatment, the 5-year survival rates for all patients diagnosed with esophageal cancer range from 15% to 20% [3]. This outcome is to some extent due to the lack of a screening approach for timely diagnosis. Indeed, ESCC patients often present at an advanced stage at the time of diagnosis when the tumor is no longer amenable to surgical resection [3, 4]. Thus, a noninvasive screening procedure aiding in early ESCC diagnosis is urgently needed and would contribute to timely treatment in ESCC.

In recent years, many studies have demonstrated that the antigenic changes of proteins in malignant cell, called tumor-associated antigens (TAAs), can be recognized by the immune system and further induce autoantibodies [5]. Importantly, autoantibodies seem to present at symptomatic stage of cancer, indicating the evaluation of autoantibodies viable for early cancer detection [5, 6].

Ezrin, a component of cell-surface structures pertaining to the member of ERM (Ezrin-Radixin-Moesin) family, acts as a linker between a number of growth factor receptors/adhesion molecules and the actin cytoskeleton [7]. Ezrin participates in cell-cell interactions and in cell adhesion to the extracellular matrix, [8, 9]. There is also evidence that Ezrin is involved in signal transduction through Rho GTPase and receptor tyrosine-kinase signaling and interacted with cellular apoptotic machinery [10, 11]. Ezrin is regarded as one of the promising key components in tumor metastasis, since it plays a role in interaction between the cell and its microenvironment, which facilitates intracellular signal transduction [12]. Ezrin is overexpressed in several kinds of cancers, which is associated with adverse outcomes [13–17]. Our previous study suggested that Ezrin might be a prognostic biomarker for ESCC [16]. Recently, Capello et al. identified autoantibodies to Ezrin as early diagnostic biomarker in pancreatic cancer [18]. Since evidences show that protein overexpression could induce humoral responses in cancer patients [19] and Ezrin proteins were found to be upregulated in ESCC (including early-stage ESCC) in comparison to adjacent normal tissue in our previous study [17], it is plausible that Ezrin might induce autoantibody production in ESCC patients.

To clarify the ability of autoantibodies against Ezrin in serum in ESCC diagnosis, we conducted enzyme-linked immunosorbent assay (ELISA) to investigate levels of autoantibodies against Ezrin in 149 ESCC patients and 98 normal controls and did western blot to confirm the results. We then explored the probable relevance between Ezrin autoantibodies and patients' clinical features.

## 2. Methods

### 2.1. Study Population

Patients with ESCC were recruited from the Cancer Hospital, Shantou University Medical College, Guangdong, China, from December 2013 to February 2015. The normal controls were from healthy individuals for physical examination at the Cancer Hospital, who had no previous and present evidence of malignant diseases based on imaging technology and had negative CEA and AFP results. The age and the sex in the patient group and the control group were matched as much as possible. Approval for the study from institutional review board of the Cancer Hospital of Shantou University Medical College and written informed consent from all patients and controls were obtained.

ESCC was defined by means of spiral computed tomography and gastroscopy and was histopathologically confirmed. Tumor stage was defined according to the seventh edition of the American Joint Committee on Cancer (AJCC) Cancer Staging Manual [20]. We classified tumors with AJCC stage 0 + I as early-stage ESCC and AJCC stage II + III + IV as advanced ESCC.

Blood specimens from normal controls and newly diagnosed ESCC patients, collected at the time of diagnosis before any treatment, were centrifuged at 1250*g* for 5 min, and the serum samples were stored at −80°C until use. Sera of patients with ESCC and normal controls were stored in a serum bank for research.

### 2.2. Expression and Purification of Recombinant Ezrin Protein

The coding sequence region for Ezrin (NM_003379) was subcloned into the pDEST17 expression vector (Invitrogen, Waltham, MA). The recombinant proteins, as described in our previous study, were expressed, purified, and analyzed [21, 22].

### 2.3. Autoantibody Detection

Two investigators (Lan Li and Ming Liu) who were blinded to clinical data of the patients and normal controls performed ELISA to measure serum autoantibodies as previously reported [21, 22]. In brief, purified Ezrin protein was diluted to 0.1 *μ*g/mL, and 100 *μ*L diluent was pipetted into each well to coat polystyrene plates. Self-made quality control samples and serum samples (at the dilution of 1 : 110) were incubated at 37°C for 1 h, as well as appropriate polyclonal anti-rabbit antibodies (Cell Signaling Technology, Cat. No. 3145) specific for capture proteins. Secondary antibodies (HRP-conjugated goat anti-human/anti-mouse IgG) were added at the dilution of 1 : 10000. At color formation step, 3,3′,5,5′-tetramethylbenzidine (50 *μ*L) and hydrogen peroxide (50 *μ*L) were add to each well. The cutoff value of positivity was defined as an OD_450 nm/630 nm_ value greater than the mean plus 2 standard deviation (s.d.) from the 98 normal controls.

Quality control for monitoring of the ELISA assay was conducted according to our previous study [21, 22].

### 2.4. Western Blot

Purified Ezrin protein was processed by electrophoretic separation on 10% SDS-PAGE and then transferred onto a polyvinylidene difluoride (PVDF) membranes by the iBlot® 2 Dry Blotting System (Thermo Fisher Scientific). The PVDF membranes were incubated in blocking buffer (TBS containing 0.05% Tween-20 (TBST) and 5% nonfat dry milk) at room temperature for 1 h and were then incubated overnight at 4°C with a 1 : 100 dilution of serum and 1 : 2000 dilution of polyclonal anti-Ezrin antibody. We used HRP-conjugated goat anti-human/anti-mouse IgG as secondary antibodies at the dilution of 1 : 5000 which was recommended by the manufacturer. Finally, we detected immunoreactive bands by using the ECL kit (Thermo Fisher Scientific), which were photographed by FluorChem 8900 (Alpha Innotech, USA).

### 2.5. Statistical Analysis

We conducted all analyses with the use of the GraphPad Prism 5 software, Microsoft Excel, and SPSS (version 17.0). The nonparametric Mann–Whitney *U* test was used to compare the Ezrin autoantibody levels between sera of normal controls and ESCC. Chi-squared tests were used for the comparison of positive rates between cancer patients and control group and to evaluate relationships of test positivity with clinicopathological features. In all tests, we considered *P* values of <0.05 (two sided) to be significant.

## 3. Results

### 3.1. Serum Level and Frequency of Autoantibodies against Ezrin in ESCC Patients

We in total obtained 247 serum samples for this study, 149 in patient group and 98 in control group. The demographics and characteristics of ESCC patients and normal controls are summarized in Table 1.

**Figure 1:**
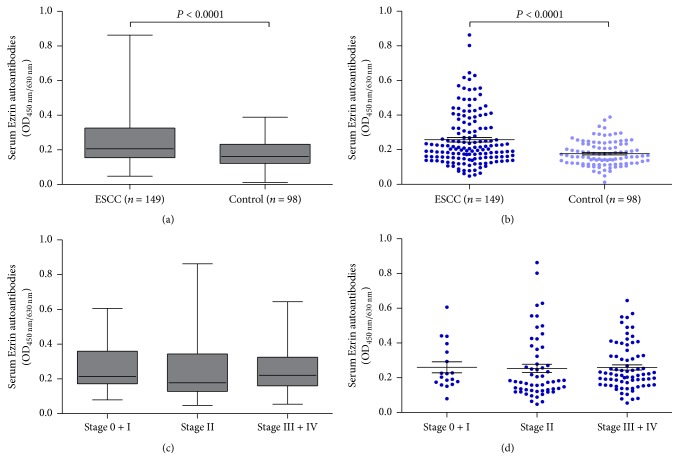
levels of serum Ezrin autoantibodies. (a) Median levels and interquartile ranges of serum Ezrin autoantibodies in ESCC patients and normal controls are illustrated by box plot and the whiskers show minimum and maximum value. (b) Scatter plots of OD values of Ezrin autoantibodies from sera of ESCC patients and normal controls. Black horizontal lines are means, and error bars are SEs. (c) Median levels and interquartile ranges of serum Ezrin autoantibodies in ESCC patients by AJCC stage are illustrated by box plot and the whiskers show minimum and maximum value. (d) Scatter plots of OD values of Ezrin autoantibodies from sera of ESCC patients by AJCC stage. Black horizontal lines are means, and error bars are SEs.

**Figure 2:**
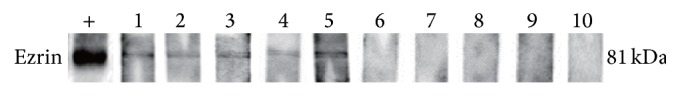
Western blotting analysis with representative sera recognizing Ezrin recombinant protein. The polyclonal anti-Ezrin antibody was used as positive control; lanes 1–5, five representative ESCC sera (with AJCC stage I, stage II, stage II, stage III, and stage III, resp.) with positive results in ELISA test have strong reactivity with Ezrin recombinant protein in western blotting analysis; lanes 6–10 are five representative normal control sera with negative reactivity.

The OD values of autoantibodies against Ezrin for ESCC patients and normal controls ranged from 0.048 to 0.863 and from 0.012 to 0.389, respectively. The mean OD ± s.d. of serum autoantibodies against Ezrin was 0.258 ± 0.153 in the 149 ESCC patients and 0.177 ± 0.071 in the 98 normal controls.  Figure 1 clearly shows that serum autoantibodies levels were higher in the ESCC patients than in normal controls (*P* < 0.0001). Box plots and Scatter plots of OD values of Ezrin autoantibodies in sera of ESCC patients by AJCC stage were also shown in Figure 1. As shown in Table 2, when we used a cutoff value of 0.319, the positive rate of autoantibody against Ezrin was 27.5% in ESCC, which was significantly higher than that in normal controls (*P* < 0.0001). We next validated the ELISA results using western blot analysis. Compared to normal serum, representative serum from ESCC patients also showed strong reactivity in western blotting (Figure 2), which were detected with positive results to Ezrin autoantibodies by ELISA method. Detection of this autoantibody assay provided a sensitivity of 27.5%, with a robust specificity of 95.9% (Table 3).

### 3.2. Diagnostic Value of Autoantibodies against Ezrin for Early ESCC and Advanced ESCC

In this study, there are 18 patients with early-stage ESCC (AJCC stage 0 + I) and 131 with advanced ESCC (AJCC stage II + III + IV), respectively. Chi-squared test showed that patients with early-stage ESCC had a higher positive rate for this autoantibody test than normal controls (*P* < 0.0001, Table 2). Similar result was also observed in advanced ESCC patients, when compared with normal controls (Table 2). The ability of autoantibodies against Ezrin to diagnose early ESCC or advanced ESCC was also evaluated (Table 3). Moreover, 1 of 4 ESCC patients (25%) who were defined to have stage 0 disease showed a positive result of autoantibody detection.

### 3.3. Effect of Clinicopathological Features on Biomarker Assay

We further assessed the differences of the Ezrin autoantibody positivity with clinical variables in ESCC patients, and we found that there were no significant correlations in assay positivity with patient age, gender, tumor size, tumor invasion depth, tumor site, histological grade, lymph node status, TNM stage or early-stage, and advanced stage groups (all *P* > 0.05, Table 4).

## 4. Discussion

ESCC lacks particular symptoms at early stage and effective noninvasive methods for screening, leading to the present situation that the detection of early ESCC is hampered [3]. Currently, the use of endoscopic screening and treatment has contributed to reduction in ESCC-associated mortality, but the early detection based on a noninvasive procedure (e.g., serum biomarker) is still imminently needed to improve the outcomes of these patients. In clinical practice, squamous cell carcinoma antigen (SCCA), carcinoembryonic antigen (CEA), and CYFRA 21-1 are used as serum tumor markers for ESCC, but low sensitivity and low specificity limit their application in the early diagnosis [23–25]. In this study, we report an ELISA assay to identify Ezrin proteins that induce a humoral response in ESCC, showing potential utility of autoantibodies against Ezrin in ESCC diagnosis.

In recent years, autoantibodies to TAAs have drawn increasing scientific interest owing to their promising value of clinical application in terms of the early detection of cancer [5, 6, 21, 22, 26–30]. As a matter of fact, biomarkers for early diagnosis of cancer require high specificity and sensitivity, but the detection of a single autoantibody appears to lack enough diagnostic power [5, 26]. An increasing number of studies have suggested that a panel of several autoantibodies, to be measured simultaneously, may overcome this issue. For example, the* EarlyCDT*-Lung test has shown that continuous addition of antigen up to seven TAAs in total increased the sensitivity to 41% in patients with lung cancer, but almost did not decrease the specificity in control samples [30]. Our study reported similar findings, which demonstrated that using a panel of six TAAs (i.e., BMI-1, PRDX6, NY-ESO-1, p53, HSP70, and MMP-7) could distinguish patients with early ESCC from normal controls, with a sensitivity/specificity of 45%/95% [21]. On the other hand, it should be emphasized that the clinical application of autoantibodies in early cancer diagnosis might rely on an optimized panel of TAAs selected with high specificity and sensitivity. We here measured autoantibodies against Ezrin in sera of patients with early-stage ESCC and normal controls resulting in 27.8% sensitivity with a robust specificity of 95.9%. The robust specificity should make this autoantibody assay possible to be selected for combining an optimized autoantibody panel which is useful in early ESCC diagnosis. When we attempted to combine Ezrin autoantibodies with autoantibodies against p53 and NY-ESO-1 which were integral to our identified autoantibody panel in previous study [21], we found that this combination presented an enhanced sensitivity with the same specificity in early ESCC diagnosis compared with either test alone (data not shown). However, the size of patients with early disease in the present study was relatively small. Thus, we need to enlarge the number of early-stage ESCC to further assess the early diagnostic ability of Ezrin autoantibody in future study.

Expression of Ezrin may be upregulated in cancer, and most probably phosphorylation of Ezrin is upregulated, which is shown to play an important role in tumor cell differentiation and metastasis [31]. Recently, a recent pancreatic cancer study reported firstly by Capello et al. provided evidence that Ezrin autoantibodies exists early in the stage of carcinogenesis [18]. These studies support the immunogenicity of Ezrin that we observed in ESCC in the present study, even though the mechanism on how autoreactive immune responses against Ezrin are induced is not clear.

In conclusion, to date this study is the first to assess the diagnostic value of serum autoantibodies against Ezrin in ESCC, indicating that the presence of autoantibodies against Ezrin is significantly associated with ESCC and may be of clinical value for ESCC. Moreover, our results reveal that autoantibody against Ezrin combined with other specific autoantibodies in esophageal cancer may shed light on a promising way to develop a highly sensitive test for early diagnosis of ESCC in the future.

## Figures and Tables

**Table 1:** Participant details and clinicopathological features.

Group	ESCC	Normal
Number	149	98
Male, *n* (%)	107 (72%)	70 (71%)
Female, *n* (%)	42 (28%)	28 (29%)
Mean age ± s.d. (years)	58 ± 9	55 ± 10
Age range (years)	41–88	34–87
Tumor invasion depth		
Tis	4	
T1 + T2	32	
T3 + T4	113	
Lymph node metastasis		
Positive	72	
Negative	77	
Histological grade		
High	40	
Medium	93	
Low	16	
TNM stage		
0	4	
I	14	
II	57	
III	44	
III	3	
Tumor size		
<5 cm	89	
≥5 cm	60	
Tumor site		
Upper thorax	17	
Middle thorax	111	
Lower thorax	21	

ESCC: esophageal squamous cell carcinoma.

**Table 2:** Positive rates of autoantibodies against Ezrin for ESCC.

Group	*N*	Positive (%)	*P* value
ESCC	149	41 (27.5%)	<0.0001
Early-stage ESCC (0 + I)	18	5 (27.8%)	<0.0001
Advanced ESCC (II + III + IV)	131	36 (27.5%)	<0.0001
Normal controls	98	4 (4.1%)	

ESCC: esophageal squamous cell carcinoma. *P* value is relative to normal controls. Statistical significance was determined using the *χ*^2^ test.

**Table 3:** Diagnostic results for autoantibodies against Ezrin in ESCC.

	Sensitivity	Specificity	PPV	NPV	PLR	NLR
ESCC versus NC	27.5%	95.9%	91.1%	46.5%	6.74	0.76
Early-stage ESCC versus NC	27.8%	95.9%	55.4%	87.9%	6.78	0.75
Advanced ESCC versus NC	27.5%	95.9%	90.0%	49.7%	6.71	0.76

ESCC: esophageal squamous cell carcinoma; NC: normal controls; NLR: negative likelihood ratio; NPV: negative predictive value; PLR: positive likelihood ratio; PPV: positive predictive value.

**Table 4:** Relationship between positive rate of the autoantibody and clinicopathologic features in ESCC patients.

Group	*n*	Positive (%)	*P* value
Patient age			
≤55	57	11 (19.3%)	0.077
>55	92	30 (32.6%)	
Patient gender			
Female	42	12 (28.6%)	0.857
Male	107	29 (27.1%)	
Tumor invasion Depth			
T1 + T2	32	10 (31.3%)	0.599
T3 + T4	113	30 (26.5%)	
Lymph node metastasis			
Positive	72	18 (25.0%)	0.506
Negative	77	23 (29.9%)	
Histological grade			
High	40	8 (20.0%)	0.274
Medium	93	30 (32.3%)	
Low	15	3 (20.0%)	
TNM stage			
I	14	4 (28.6%)	0.234
II	57	15 (26.3%)	
III	44	20 (45.5%)	
IV	3	1 (33.3%)	
Tumor size			
<5 cm	89	27 (30.3%)	0.348
≥5 cm	60	14 (23.3%)	
Tumor Site			
Upper thorax	17	3 (17.6%)	0.364
Middle thorax	111	30 (27.0%)	
Lower thorax	21	8 (38.1%)	
Early-stage versus advanced-stage			
Early-stage	18	5 (27.8%)	0.979
Advanced-stage	131	36 (27.5%)	

Statistical significance was determined by means of Chi-squared test.
